# Laboratory Study of Deformation Behaviour of Two New Reinforcing Polymeric TSLs and Their Potential Application in Deep Underground Coal Mine

**DOI:** 10.3390/polym13132205

**Published:** 2021-07-03

**Authors:** Han Liang, Jun Han, Chen Cao, Shuangwen Ma

**Affiliations:** 1 College of Mining, Liaoning Technical University, Fuxin 123000, China; hanj_lntu@163.com (J.H.); cc752@uowmail.edu.au (C.C.); maswen3935@163.com (S.M.); 2 Liaoning Province Coal Resources Safety Mining and Clean Utilization Engineering Research Center, Fuxin 123000, China; 3 EIS, CME, University of Wollongong, Wollongong, NSW 2522, Australia

**Keywords:** reactive TSL, reinforcing TSL, mechanical behaviours, experimental study

## Abstract

Thin spray-on liner (TSL) is a surface protection technology used by spraying a polymer film, which is widely used for mine airtightness and waterproofing. A reinforcing TSL can replace steel mesh, which is a new method for roadway support. This paper reviews the development of a reinforcing TSL. Considering the deterioration of geological conditions in deep underground mining and the demand for reinforcing automation, two kinds of polymeric reinforcing TSL (RPTSL) materials are developed. The mechanical characteristics of the new TSL materials are studied experimentally. Results show that the average compressive strength, tensile strength, cohesion, and internal friction angle of the two TSL materials are 52 and 32 MPa, 12 and 8 MPa, 6.2 and 17.2 MPa, and 33.6° and 25.9°, respectively. The bonding strength between the two materials and coal is greater than the tensile strength of coal itself, and the mechanical properties of the material for comparison are lower than those of both materials. Based on the TSL support mechanism, we examine the application of the two TSL materials to the mining environment and compare the mechanical properties of polymer materials and cement-based materials. The advantages of polymer materials include versatile mechanical properties, good adhesion, and high early strength. This study provides a new support material to replace steel mesh for roadway surface support, which satisfies the needs of different surface support designs under complex geological conditions, and promotes the automation of roadway support.

## 1. Introduction

Thin spray-on liner (TSL) is a practical technology for surface protection used by spraying a polymer film. Since the polymer spray coating is ductile, it has been widely used for waterproofing and airtightness in sprayed concrete projects since the 1980s.

Reinforcing TSL refers to the utilization of a TSL to replace steel mesh for roadway support. The installation of steel mesh in underground tunnels is labour intensive, which is difficult to realize mechanization and automation. The main purpose of the early research on the reinforcing TSL is to solve labour shortage and accelerate excavation speed. Villaescusa [1] used a TSL instead of steel mesh and conducted field tests in two hard rock roadways from 1999 to 2000.

A reinforcing TSL possesses better support performance than steel mesh. In terms of surface protection, the TSL not only bonds the surface of the surrounding rock, but also can penetrate the surface cracks so that the rock mass with developed surface cracks becomes relatively integrated [2]. Regarding the reinforcing mechanism of the TSL, Stacey [3] divided the reinforcing effect of the TSL into three stages, namely, bonding stage, deformation stage, and shell effect stage. At the initial stage of spray, the TSL bonds the surface and surface cracks of the surrounding rock, and the bonding strength between the TSL spray layer and the surrounding rock is an important parameter to quantify its support performance. After the surrounding rock begins to deform, the TSL enters the deformation stage, and the tensile strength and the shear resistance together determine the coupling level between the spray layer and the surrounding rock. After the surrounding rock is further deformed or even debonding from the TSL material, the spray layer enters the thin shell effect stage. The bearing capacity of the shell structure mainly depends on the compressive strength of the material. Therefore, the compressive, tensile, shear, and bonding behaviours of TSL materials with a surrounding rock are the most important mechanical parameters to examine their support performance.

In the past 10 years, the main researchers on reinforcing TSL include the University of Wollongong in Australia, the Middle East University of Science and Technology in Turkey, and the Jinshan University in South Africa. Chinese research institutions include the Academy of Coal Science, Shandong University of Science and Technology, and Liaoning Technical University. The main research focuses on material development and mechanical performance evaluation, including experimental research on compressive, tensile, shear, and bond strength. Yilmaz [4] made 20 “dog bone” specimens from 35 samples of 17 TSL manufacturers in 2010 to test the effect of curing time on tensile strength and compared them with concrete samples. Results showed that the tensile strength of most TSL materials increased with curing time. After 28 days of curing, the tensile strength ranged from 0.5 to 7.8 MPa. The tensile strength of the concrete sample for comparison was about 0.5 MPa. ToughSkin developed by the University of Wollongong is currently leading the research and development of reinforcing TSL materials. The average compressive strength of the 40 mm-long cubic sample of the developed TSL material was 77.7 MPa. Based on the punching shear test of the 250 × 85 × 5 mm plate sample, the measured average shear strength of the material was about 33 MPa [5]; the bonding strength with coal was 0.76 MPa [6]. The material is violently ejected with small fragments when it is crushed under compression. In order to improve the material resilience, subsequent studies have incorporated glass fibres in the TSL material. In 2019, Ozturk [7] developed two TSL materials, flexible and rigid. The compressive strengths of the two TSL materials (cylindrical specimens with a diameter of 25 mm and a length of 50 mm after curing for 28 days) were 34.0 and 3.7 MPa, and the tensile strengths were 5.1 and 5.3 MPa, respectively [8]. Zhang [9] developed two TSL materials in 2017 with tensile strengths of 3.3 and 7.6 MPa, respectively. The tensile strength of the German-produced TSL was 7.9 MPa. Chen [10] experimentally studied the bonding strength of a nonreactive TSL material with four types of rocks (granite, sandstone, concrete, and coal) in 2020. The results showed that bonding strength was most stable under a coating thickness of 5 mm, and the nominal compressive and tensile strengths of the experimental materials were 12.4 and 2.7 MPa, respectively. A few studies have tested the support performance of the TSL, such as [11,12,13]. The preliminary experimental research mostly followed the American Society of Testing Materials (ASTM) standards [14] but with slight differences.

In 2020, the Chinese government issued a statement that the intelligence of high-production coal mines will be basically realized by 2025, and the intelligence of all coal mines will be basically realized by 2035 [15,16,17,18]. Moreover, as coal mines go deeper, roadway support faces higher ground stress and larger deformation of the surrounding rock. The mesh support operation mainly relies on labour work, which is time-consuming and laborious. Therefore, the development of a new support method is a shortcut to the intelligent construction of bolting [19,20].

Although considerable progress has been made in the research and development of reinforcing TSL materials recently [7], there are still too few TSL products to meet the reinforcing requirements of deep mining with complex geological conditions. The main reason for the relatively nonversatile performance of a reinforcing TSL is the use of nonreactive materials. The main purpose of nonreactive materials is to reduce cost, but the mechanical properties are rather limited. A polymer-based TSL owns the advantages of high early strength and multiple combinations of mechanical properties. This type of material has been rarely studied, and it has great potential in deep underground resource extraction. We have developed two polymer TSL materials with different substate materials. Here, we conduct experimental studies on their mechanical properties and compare them with the reactive sealing material, Malisan (MLS), which is commonly used in mines to provide a basis for the application in deep underground roadway spray support.

## 2. Materials and Methods

**Figure 1:**
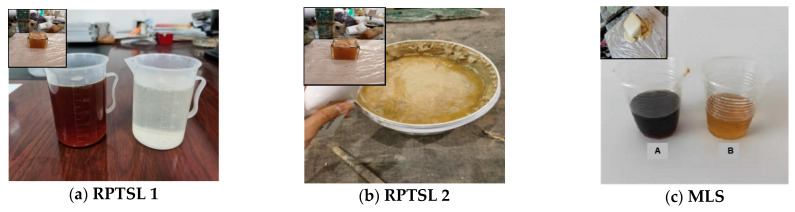
Three TSL materials tested.

We experimentally measured the compressive strength, shear strength, tensile strength, and adhesion with the coal of three polymer materials. RPTSL 1 is a modified polyurethane substate material (Figure 1a), RPTSL 2 is an epoxy-based material (Figure 1b), and MLS is a polymer sealing material used in a coal mine in Tangshan, China, utilised to make a comparison with the former two (Figure 1c). During sample preparation, there was no obvious foaming and heating in the chemical reaction of RPTSL 1 (Figure 1a small image), RPTSL 2 produced a small amount of heat (Figure 1b small image), and MLS experienced foaming and heating (Figure 1c small image). All samples were tested after curing for 7 days at room temperature.

The mechanical property tests include uniaxial compressive, shearing, tensile, and bonding strength tests. The number of testing samples in each experiment is shown in Table 1.

### 2.1. Design of Uniaxial Compression Test

**Figure 2:**
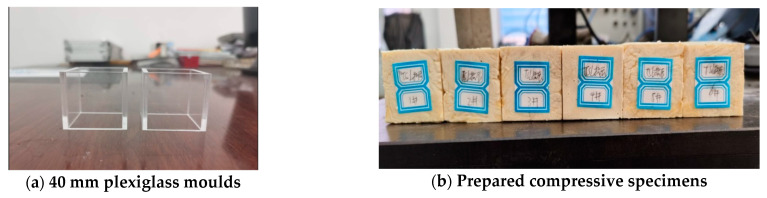
Compression mould and sample.

For the compression with other TSL research studies in the literature, ASTM standard was used in the uniaxial compressive test. Plexiglass moulds were used to prepare the testing specimens, and the TSL sample size was 40 mm in cubes, as shown in Figure 2. Six samples were prepare for each kind of TSL material.

**Figure 3:**
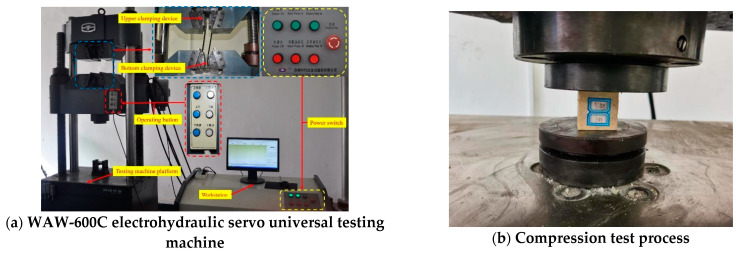
Compression test instrument and process.

The experimental machine was a WAW-600C computer-controlled electrohydraulic servo universal testing machine (Time Shijin, Jinan, China), as shown in Figure 3a. The loading mode was displacement control with a loading rate of 1 mm/min. The testing arrangement is shown in Figure 3b. As the available strain gauge and extensometer can only be used for a standard rock mechanical testing sample, only axial load and displacement were measured, and the stress–strain curve was then calculated.

### 2.2. Design of Variable Angle Shear Test

**Figure 4:**
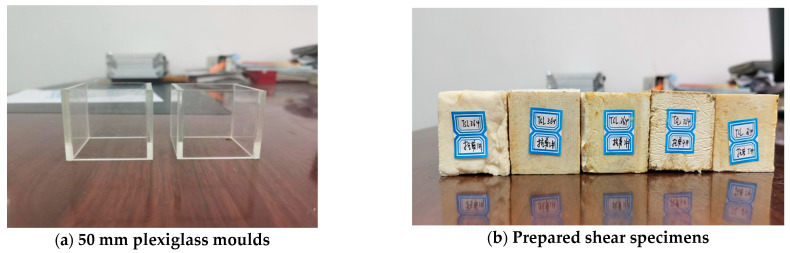
Shear moulds and TSL samples.

A variable angle testing method was employed for the shearing test due to the machinery availability. According to rock mechanic testing standard, 50 mm cube samples were prepared using corresponding plexiglass moulds, as shown in Figure 4a. There were 5 shearing angles in the test; thus, 5 samples were prepared for each TSL material, as shown in Figure 4b.

**Figure 5:**
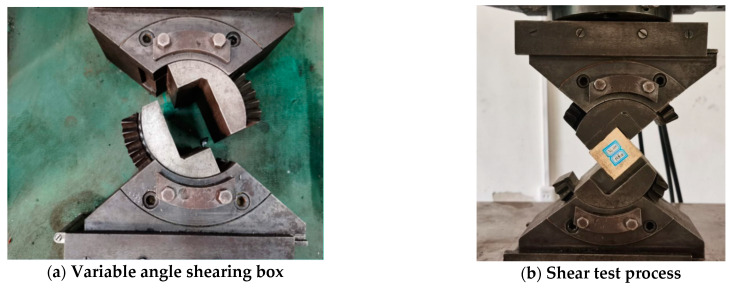
Shear test instrument and process.

A WAW-600C experimental machine and a variable angle shearing box were used in the test, as shown in Figure 5a. The shear angles were 42°, 50°, 58, 66°, and 74°, and the loading rate was 1 mm/min. The testing procedure is shown in Figure 5b. The axial load and displacement were collected, and the normal stress and shear stress of the shearing plane can be calculated.

### 2.3. Design of Tensile Test

**Figure 6:**
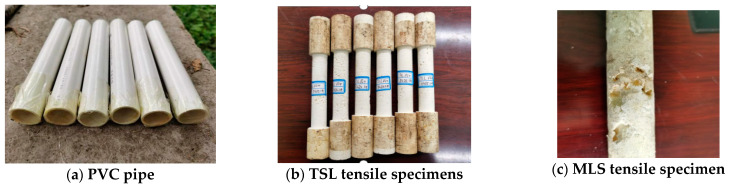
Tensile moulds and samples.

In standard ASTM, the tensile testing sample is a 4 mm thick dog bone. However, in this study, the tensile testing samples were designed as a 20 mm cylinder to investigate the size and distribution of the bubbles generated in the chemical reaction. PVC tubes with inner Φ30 mm and 200 mm lengths were used to prepare the TSL samples, as shown in Figure 6a. The dimensions of the clamping end and the test section of the specimen were Φ30 × 50 mm and Φ20 × 100 mm, respectively, as shown in Figure 6b. Six tensile samples were prepared for TSL 1 and 2 materials, but only 3 samples were prepared using MLS. It was found that the MLS samples were loose due to the existence of a huge number of bubbles generated in the chemical reaction, as shown in Figure 6c. In the test, the MLS samples could not be clamped firmly by the testing machine, so only TSL 1 and 2 samples were tested.

**Figure 7:**
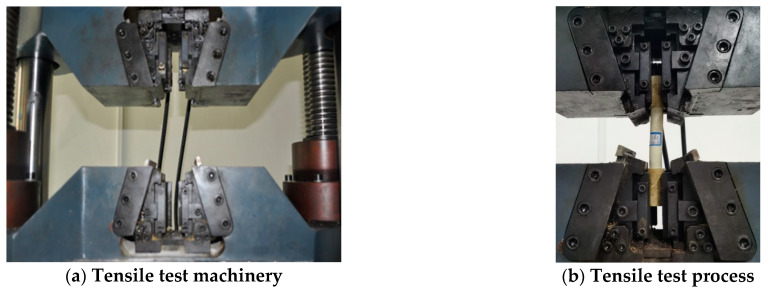
Tensile test instrument and process.

The testing machine was WAW-600C with stretching tools, as shown in Figure 7a. The loading rate was 1 mm/min, and the testing procedure is shown in Figure 7b. The axial load and displacement were collected, and the stress–strain curve was calculated.

### 2.4. Design of Bonding Strength Test

The bonding test was designed based on the bonding mechanism of the TSL with a surrounding rock developed by Yilmaz [21]. In the test, the bonding strength of TSLs with lignite was measured. The average tensile strength of the lignite was measured to be 1.12 MPa via the Brazilian tests.

**Figure 8:**
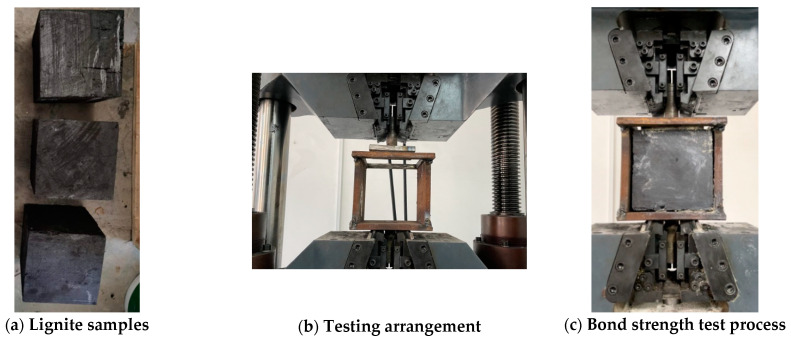
TSL–lignite bonding test.

In order to measure the bonding strength between the TSL material and coal, the lignite was cut into 150 mm cubes, as shown in Figure 8a. A steel framework was constructed to hold the lignite tube, as shown in Figure 8b. The TSL was sprayed on the lignite in an area of 120 × 120 mm, and the coating thickness was 5 mm. The bonding samples were cured for 7 days at room temperature. Then, they were put into the steel framework and glued with one steel pulling plate. The testing procedure is shown in Figure 8c. The loading rate was 1 mm/min.

## 3. Results and Analysis

**Figure 9:**
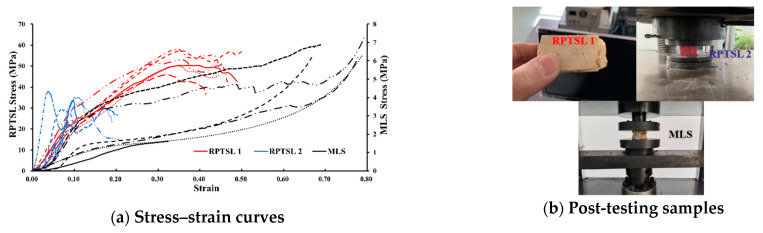
Compressive testing result.

Figure 9a shows the stress–strain curves of three types of material under compression; the right Y-axis is for MLS. The performance under compression of TSL 1 was much better than that of TSL 2. In the compressive test, TSL 1 was slightly damaged in the strain range of 0.3–0.4, and TSL 2 had fragments bounced out at a strain of about 0.1, indicating brittle failure at this stage, whereas MLS displayed a flexible behaviour under compression, and its strain was higher than 0.8 with a compressive strength greater than 6 MPa. The post-testing specimens of each tested material are shown in Figure 9b. The fractured section of TSL 1 was rougher compared with that of TSL 2.

**Figure 10:**
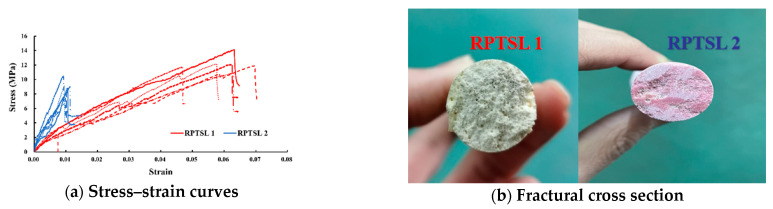
Tensile testing result.

The tensile testing results are shown in Figure 10. As the MLS specimen could not be clamped by the testing machine, only TSL samples were tested. The elongation at tension failure of RPTSL 1 was five times that of RPTSL 2. The fractured section of the tested TSL materials is shown in Figure 10b.

**Figure 11:**
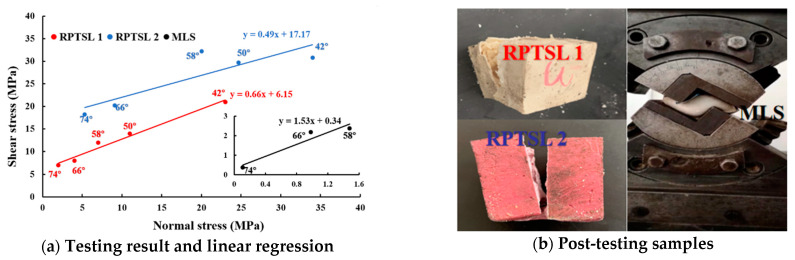
Shearing test result.

The shearing test results are shown in Figure 11; the minor image shows the shearing performance of MLS. The shearing properties of each material were obtained via linear regression, as shown by the formulars. In the shear test, the deformation range of MLS at shear angles of 42° and 50° was beyond the measurement range of the machine (Figure 11b), and thus the shearing properties were obtained based on three testing results.

**Figure 12:**
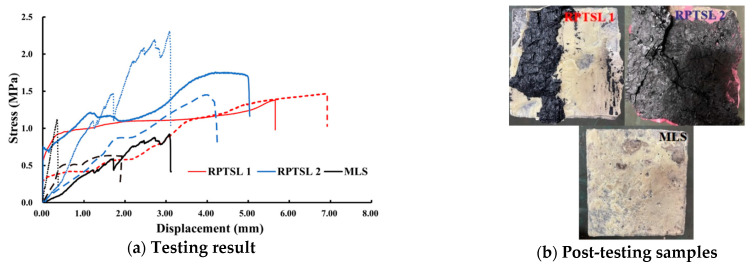
Bonding test result.

For the adhesive test, the bonding performance of the tested materials with lignite is shown in Figure 12. One TSL 1 testing was not successful, and only eight curves were obtained. Figure 12b shows the typical failure surface of the specimens. For two RPTSLs, it suggests that the bonding strength between the studied material and coal is equal to or higher than the tensile strength of the coal; for MLS, its bonding strength is greater than the tensile strength of itself, but less than the tensile strength of the coal.

All testing results are summarized in Table 2. It shows that the average compressive strengths of the two test materials are higher than 30 MPa, which is 5 to 8 times that of MLS. Comparison between the two materials shows that the average compressive and tensile strengths of RPTSL 1 are greater than those of RPTSL 2, and the ductility under compression and tension is also more remarkable than that of RPTSL 2. Specifically, the average strains of RPTSL 1 and RPTSL 2 at the peak compressive strength are 0.36 and 0.09, respectively. The compressive and tensile performances of RPTSL 1 are better than those of RPTSL 2, which demonstrates that the shell structure formed has higher support capacity. RPTSL 2 has greater shear resistance and bonding strength than RPTSL 1. It can better bond the surface of the surrounding rock as a whole at the initial stage of spraying and mitigate the early deformation. Meanwhile, the high shear strength benefits the surrounding rock control. The roadway deformation caused by the dislocation has a good ability in controlling the deformation of the surrounding jointed rock masses.

Table 2 also shows a comparison between the mechanical performances of the materials developed in this study and other reinforcing TSL materials in the literature, mainly polymer-based ToughSkin [5,6] and one recently developed cement-based TSL [10]. Result indicates that the overall mechanical properties of the reinforcing TSL developed by the University of Wollongong are still superior. Optimization of the ratio to improve the overall material performance is the focus of subsequent research.

Comparing the two polymer TSL materials in this study with the nonreactive flexible and rigid TSL materials developed by the Middle East University of Science and Technology in Turkey [7,8], it is found that the compressive and tensile strengths of the two polymer materials in our study are higher. The advantage of the nonreactive flexible TSL material lies in its large deformation, which exceeds the two materials in this study. However, its strength is smaller than 4 MPa at 50% of its total deformation, which indicates that the increase in the deformation of the surrounding rock will lead to a significant degradation in the performance of surface protection.

## 4. Conclusions

To cope with the complex geological conditions of deep underground resource mining and accelerate automatic mining, two polymer TSL materials were developed to replace steel mesh support. The deformation of the two TSL materials was experimentally studied, and the following conclusions are made:

First, experimental studies on the mechanical properties of two experimented TSL materials show that the average compressive strength, tensile strength, cohesion, and internal friction angle of RPTSL 1 are 52 MPa, 12 MPa, 6.2 MPa, and 33.6°, respectively. The average compressive strength, tensile strength, cohesion, and internal friction angle of RPTSL 1 are 32 MPa, 8 MPa, 17.2 MPa, and 25.9°, respectively. Both of the bonding strengths of the two materials and coal are higher than the tensile strength of the coal itself. The mechanical properties of the material for comparison are lower than those of the new TSL materials.

Second, comparative analysis of the two experimental materials demonstrates that RPTSL 1 has higher compressive strength, tensile strength, and ductility, and the formed shell structure has better supporting capacity. RPTSL 2 has high shear resistance and bonding strength, which benefits bonding the surrounding rock surface as a whole in the initial spray stage and mitigates the dislocation and deformation of the surrounding rock. The two materials can meet the reinforcing requirements of different mining conditions in deep underground mining.

Third, compared with the cement-based nonreactive TSL material, the experimented polymer TSL material has obvious advantages in high early strength. In the construction of intelligent mines, the automatic control of RPTSL is simple and reliable, and it is a new support material that accelerates the construction of unmanned heading faces.

## Figures and Tables

**Table 1:** Experiment and testing number.

Experiment	Material	Number
Uniaxial compressive test	RPTSL 1	6
	RPTSL 2	6
	MLS	6
Variable angle shear test	RPTSL 1	5
	RPTSL 2	5
	MLS	5
Tensile test	RPTSL 1	6
	RPTSL 2	6
Bonding strength test	RPTSL 1	3
	RPTSL 2	3
	MLS	3

**Table 2:** Summary of experimental results.

Material	Sample	Compression					Tension					Shear			Bonding		
		Strength (MPa)	Deformation (mm)	Ave Strength (SD) (MPa)	Ave Peak Deformation (mm)	Comparison (%)	Strength (MPa)	Elongation (%)	Ave Strength (SD) (MPa)	Ave Elongation (%)	Comparison (%)	Cohesion (MPa)	Friction Angle (°)	Comparison (%)	Strength (MPa)	Ave Strength (SD) (MPa)	Comparison (%)
RPTSL 1	1#	50.39	14.69	52.48 (4.60)	13.91	Decrease 40 [5,6]	14.14	6.16	12.15 (1.07)	6.04	Decrease 40 [5,6]	6.15	33.60	Decrease 50 [5,6]	1.38	1.63 (0.36)	Increase 114 [5,6]
	2#	50.20	13.76				12.14	5.75									
	3#	57.26	14.15				11.90	6.96							2.05		
	4#	58.04	13.82			Increase 320 [10]	11.75	4.68			Increase 350 [10]			/			Increase 8 [10]
	5#	52.97	14.22				10.89	6.26							1.47		
	6#	45.99	12.80				12.08	6.45									
RPTSL 2	1#	33.82	3.90	33.13 (3.22)	3.97	Decrease 134 [5,6]	8.81	1.28	8.94 (0.92)	1.14	Decrease 90 [5,6]	17.17	25.89	Decrease 6 [5,6]	1.76	1.85 (0.42)	Increase 143 [5,6]
	2#	35.23	4.36				9.17	1.02									
	3#	29.62	2.82				7.96	1.48							2.31		
	4#	38.01	1.46			Increase 167 [10]	10.55	0.92			Increase 231 [10]			/			Increase 23 [10]
	5#	32.09	4.38				9.02	1.13							1.49		
	6#	30.00	6.88				8.14	1.01									
MLS	1#	>2	12.97	>6(/)	26.98	/	/	/	/(/)	/	/	0.34	56.79	/	0.60	0.88 (0.27)	/
	2#	>6	31.51														
	3#	>6	26.73												0.92		
	4#	>6	31.23														
	5#	>7	31.88												1.13		
	6#	>6	27.55														

## Data Availability

All data of this article has been included in this manuscript.
